# Continuous Determination of Glucose Using a Membraneless, Microfluidic Enzymatic Biofuel Cell

**DOI:** 10.3390/mi11121129

**Published:** 2020-12-20

**Authors:** Haroon Khan, Jin Ho Choi, Asad Ullah, Young Ho Kim, Gyu Man Kim

**Affiliations:** 1 School of Mechanical Engineering, Kyungpook National University, Daegu 41566, Korea; mechy_365@yahoo.com (H.K.); sasadullah84@gmail.com (A.U.); 2 Insung Medical Co., Daegu-Gyeongbuk Medical Innovation Foundation, Daegu 41071, Korea; chlwlsgh01@gmail.com; 3 Medical Device Development Center, Daegu-Gyeongbuk Medical Innovation Foundation, Daegu 41061, Korea; yhkim@dgmif.re.kr

**Keywords:** continuous glucose determination, membraneless, enzymatic biofuel cell

## Abstract

In this article, we describe an enzyme-based, membraneless, microfluidic biofuel cell for the continuous determination of glucose using electrochemical power generation as a transducing signal. Enzymes were immobilized on multi-walled carbon nanotube (MWCNT) electrodes placed parallel to the co-laminar flow in a Y-shaped microchannel. The microchannel was produced with polydimethylsiloxane (PDMS) using soft lithography, while the MWCNT electrodes were replicated via a PDMS stencil on indium tin oxide (ITO) glass. Moreover, the electrodes were modified with glucose oxidase and laccase by direct covalent bonding. The device was studied at different MWCNT deposition amounts and electrolyte flow rates to achieve optimum settings. The experimental results demonstrated that glucose could be determined linearly up to a concentration of 4 mM at a sensitivity of 31 mV∙mM^−1^cm^−2^.

## 1. Introduction

Enzyme-based electrochemical biosensors, which use enzymes for signal transduction, have been extensively studied due to their high specificity and sensitivity toward their target analyte [1,2]. Typical analytes including glucose, lactate, cholesterol, glutamate, and urea, play a vital role in our body’s biochemistry, thereby implying the need for the proper monitoring of their concentrations. To achieve that, a particular set of enzymes is needed that catalyze specific biochemical reactions to measure the corresponding analytes in complex biological fluids. Glucose oxidase (GOx) is the most widely employed enzyme for glucose oxidation in the area of biosensors and biofuel cells due to its extremely specific catalytic activity for glucose [3,4]. Ever since the first demonstration of an enzymatic glucose sensor in the early 1960s, research interest has grown considerably, thereby achieving many advancements in the field of biosensors [5]. This progress has been reported in terms of the use of advanced materials, electrode design, enzyme immobilization strategies, analytical measurement methods, miniaturization techniques, etc. [6,7,8,9,10]. To get a high specific surface area, antifouling properties, and high conductivity, carbon nanotubes (CNTs) provide a versatile tool to construct bioelectrodes for electrochemical applications. Due to their high three-dimensional electroactive area that increases the surface concentration of both the enzymes and the redox mediators, CNTs have been the choice of scientists to utilize in enzymatic biofuel cells (EBFCs) [6].

EBFCs that use enzymes as the biocatalyst to generate electric power have gained significant research interest due to their operation in normal conditions [11,12]. In glucose/oxygen biofuel cells, glucose is oxidized at the anode while oxygen is reduced at the cathode to produce electricity. The produced power output of the EBFC can be used as an analytical signal that is directly proportional to the glucose concentration, thereby eliminating the need for an additional power source [13,14,15]. Such devices are termed self-powered glucose sensors (SPGSs). They have an extremely simple design due to a two-electrode setup instead of three electrodes, which is very beneficial for device miniaturization. Since the first demonstration of self-powered biosensors by Willner and Katz based on glucose [16], exciting progress has been made by researchers in the fields of both implantable and wearable diagnostics [17]. This concept was then expanded to other analytes such as fructose, lactate, ascorbic acid, and cholesterol. Additionally, different approaches have been implemented for monitoring these biomarkers; for instance, many authors have described microneedle- and paper-based SPGSs [18,19,20,21]. However, there has not yet been any research published on microfluidic, membraneless EBFCs as self-powered biosensors.

The integration of biosensors into lab-on-chip technology using microfluidics has resulted in robust analytical tools, thereby providing integrated and miniaturized substitutes for conventional laboratory methods [22,23]. Previously, we reported the fabrication of a Y-shaped microfluidic EBFC for power generation [24]. In this work, we applied that microfluidic EBFC as a proof-of-concept self-powered biosensor for the electrochemical detection of glucose using enzymes as bio-recognition elements. To the best of our knowledge, this is the first time that a co-laminar, flow-based, and membraneless, microfluidic chip has been used for the continuous detection of glucose. The proposed microfluidic EBFC as a self-powered biosensor showed high sensitivity and high recovery rates, which is advantageous for continuous glucose determination. From our test results, the microfluidic device can be used for glucose detection within a linear range of up to 4 mM.

## 2. Materials and Methods

### 2.1. Chemicals

The following chemicals were purchased from Sigma-Aldrich and were used without further purification: glucose oxidase (GOx) from *Aspergillus niger* (128.2 U∙mg^−1^ solid), laccase from *Trametes versicolor* (12.9 U∙mg^−1^ solid), N-hydroxysulfosuccinimide (NHS; 98%), N-ethyl-N’(3-dimethylaminopropyl)carbodiimide hydrochloride (EDC; 98%), 2,2′-azino-bis(3-ethylbenzothiazoline-6-sulfonic acid) (ABTS), multi-walled carbon nanotubes (MWCNTs; carboxylic acid-functionalized), sodium perchlorate (NaClO_4_, 98%), D-(+)-glucose, ferrocenemethanol (FcCH_2_OH), and indium tin oxide (ITO) glass (15 Ω.sq^−1^). Uric acid (99%), L-(+)-ascorbic acid (99+%), and 4-acetamidophenol (98%) were purchased from Alfa Aesar, South Korea. The aqueous solutions were prepared in deionized (DI) water. Polydimethylsiloxane (PDMS; Sylgard 184, Dow Corning, Midland, MI, USA) was mixed in a 10:1 ratio of base and curing agent. All analytical experiments were performed in a sodium phosphate buffer prepared with 50 mM NaH_2_PO_4_ and 50 mM Na_2_HPO_4_; the pH was adjusted by their proper mixing.

### 2.2. Biosensing Principle

The working principle of the electrochemical microfluidic EBFC is based on the enzymatic reactions of GOx and laccase at the anode and cathode, respectively, according to the following equations:

C_6_H_12_O_6_ → C_6_H_10_O_6_ + 2H^+^ + 2e^−^

O_2_ + 4H^+^ + 4e^−^ → 2H_2_O

**Figure 1:**
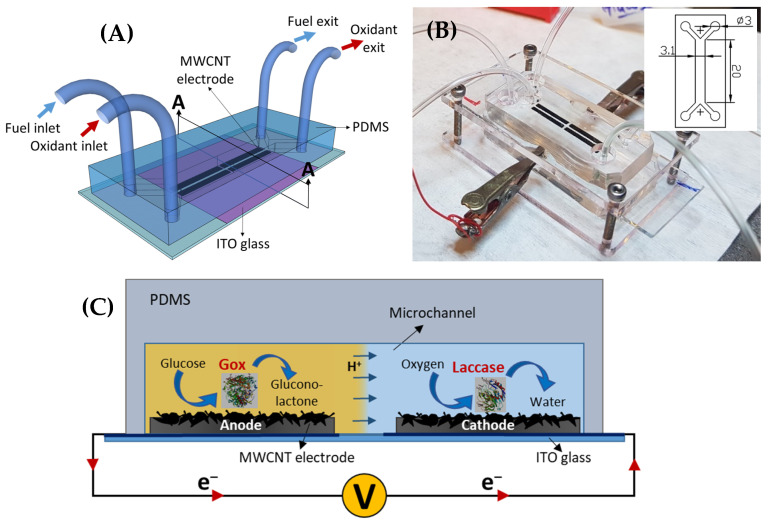
A schematic (**A**) and optical (**B**) image of the membraneless, microfluidic enzymatic biofuel cell (EBFC) (Inset shows microchannel dimensions in mm). (**C**) Schematic presentation of cross-section (A–A) illustrating the biosensing mechanism of the microfluidic EBFC.

The EBFC operation is described in the schematic diagram in more detail in Figure 1c. The fuel and oxidant flow in the microchannel in a co-laminar fashion without convective mixing, thereby eliminating the need for a proton exchange membrane [25]. Glucose is oxidized to gluconolactone by GOx immobilized at the anode. The electrons are efficiently transferred to the anode surface using FcCH_2_OH as a mediator. These electrons flow towards the cathode side through the external circuit. Protons produced by the oxidation reaction travel towards the cathode side through the co-laminar flow interface between the electrolytes in the microchannel. Likewise, oxygen is reduced to water by using laccase as an enzyme biocatalyst immobilized at the cathode. The mediator, ABTS, is used to shuttle the reducing equivalents from the cathode to the active sites of laccase. In this way, the microfluidic device generates power, which is a function of glucose concentration in the flow stream.

### 2.3. Bioelectrode Preparation

Before MWCNT electrode fabrication, the ITO glass (used as a conductive substrate) was wet-etched in concentrated HCl using a patterned AZ-1512 photoresist made by photolithography. Additionally, PDMS stencils, which were created by spin-coating the PDMS solution onto the master mold at 800 rpm for 30 s followed by blowing with a gentle nitrogen stream and curing at 90 °C for 1 h, were used to produce MWCNT electrodes on the ITO glass.

The master molds for both the stencil and microchannel were fabricated via photolithography using the negative SU-8 2100 photoresist. The thicknesses of the master molds were 250 and 120 µm for the stencil and microchannel, respectively; the whole procedure of making them was discussed in detail in our previous work [24]. Both master molds were salinized with tridecaflouro-1, 1, 2, 2-tetrahydroocty1-1-trichlorosilane in a desiccator before use.

To prepare the MWCNT electrodes, the etched ITO glass was cleaned with DI water and placed in 2% 3-amino-propyltriethoxysilane in acetone for 1 h and then dehydrated on a 120 °C hot plate for 1 h. The stencil was manually aligned on the ITO glass in such a way that the conduction gap between the electrodes was covered. A suspension of 1 mg/mL MWCNT in DI water was sonicated for 1 h, and 20 µL of this suspension was pipetted onto the stencil on ITO glass placed on a hot plate at 100 °C and allowed to evaporate. This dispensing process was repeated until the amount of MWCNTs reached the desired value. For example, the suspension was pipetted eight times to obtain a uniform surface of 0.4 mg/cm^2^ MWCNT.

For the covalent attachment of enzymes to the carboxylic acid-functionalized (COOH-) MWCNT electrodes, NHS was used to activate the carboxyl groups of MWCNTs using EDC coupling chemistry via amide bonds [26]. For this, 2 mL of a phosphate buffer (50 mM; pH 7) containing 30 mM of EDC and 90 mM of NHS was pipetted onto the surface of the electrodes and allowed to react for 1 h at room temperature. After drying gently with nitrogen gas, the anode and cathode were coated with GOx and laccase (5 mg∙mL^−1^ in a 50 mM phosphate buffer at pH 7), respectively, and allowed to react for 12 h. The electrodes were then thoroughly rinsed with the phosphate buffer (50 mM; pH 7) before use.

### 2.4. Microfluidic EBFC Fabrication

Schematic and optical images of the microfluidic device are shown in Figure 1. The microchannel was produced via PDMS casting using soft lithography. The height of the microchannel was 120 µm, for which the silicon master mold was fabricated using a negative SU-8 photoresist, as already discussed in detail in the previous section. After the master mold was salinized, the degassed PDMS solution (10:1 of base and curing agent) was poured onto it and cured at 90 °C (for at least 2 h). The PDMS replica was then peeled off from the mold and punched with a sharpened biopsy punch (1.5 mm) to produce the inlet and outlet for Tygon tubes. Finally, the PDMS microchannel was manually aligned and attached to the ITO glass containing the covalently attached enzyme-MWCNT bioelectrodes and clamped in an acryl holder to avoid any leakage.

### 2.5. Electrochemical Measurements

All the electrochemical experiments were performed using a Bio-Logic Science Instruments SP-50 model potentiostat supplied with the EC-Lab^®^ software package, and all the measurements were carried out at room temperature. For cyclic voltammetry (CV) analysis, a three-electrodes setup was used; an Ag/AgCl (saturated KCl.) electrode was the reference, a platinum wire was the counter, and a carboxyl-MWCNT (on ITO glass) electrode was used as the working electrode. The anolyte stream consisted of a sodium phosphate buffer (50 mM; pH 7) containing 100 mM NaClO_4_, 1 mM FcCH_2_OH, and D (+)-glucose (varying concentrations), while the catholyte solution contained a sodium phosphate buffer (50 mM; pH 5) with 100 mM NaClO_4_ and 2 mM ABTS. Since the redox-active site of Gox, which is flavin adenine dinucleotide (FAD), is deeply buried within a protective protein shell, the realizing of direct electron transfer (DET) is very difficult [27]. Therefore, different mediators are utilized for efficient electrochemical communication between a redox center and electrodes to ensure accurate glucose detection in biosensors [28,29]. This choice of mediators, i.e., FcCH_2_OH at the anode and ABTS at the cathode, was made since their redox potential lies between the redox potential of the enzymes and that of the electrodes [30,31]. A D (+)-glucose stock solution was prepared at least 24 h before the experiment to establish an anomeric equilibrium between α and β cyclic forms of D (+)-glucose.

## 3. Results and Discussion

### 3.1. Electrocatalytic Oxidation of Glucose at GOx-Modified MWCNT Bioanode

**Figure 2:**
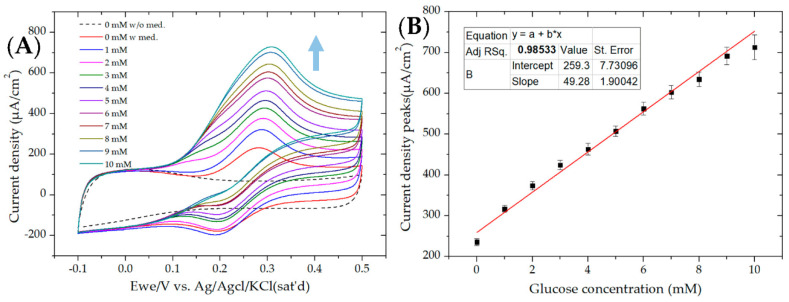
(**A**) Cyclic voltammograms of a glucose oxidase (GOx)-modified bioanode during the successive addition of varying glucose concentrations at 0 mM without a mediator (dashed curve) and 0–10 mM with a mediator (solid curves) in a sodium phosphate buffer (50 mM; pH 7) at a scan rate of 10 mV/sec. The arrow indicates increasing glucose concentration. (**B**) Corresponding calibration curve of the current density peaks at different glucose concentrations with a coefficient of correlation of R^2^ = 0.985.

Before analyzing microfluidic EBFC for glucose monitoring, the electrocatalytic behavior of the GOx-modified bioanode was explored via the CV test at varying glucose concentrations ranging from 0 to 10 mM, as shown in Figure 2A. The active surface area of bioanode fabricated for CV analysis was 1 cm^2^. The bioanode was scanned between −0.1 and +0.5 V versus Ag/AgCl in a 50 mM phosphate buffer (pH 7) containing 100 mM NaClO_4_ and 1 mM FcCH_2_OH. CV was performed at a scan rate of 10 mV/sec.

It could be seen that without both the mediator and glucose, there was no oxidation and reduction peaks (dashed CV curve in Figure 2A). However, when mediator was used at 0 mM glucose, a pair of well-reversible redox peaks with a formal potential of 0.24 V was observed, and this was assigned to one-electron reversible redox reaction of ferrocene–ferrocenium ion (Fc/Fc^+^) [32,33]. Additionally, with a glucose concentration of 1–10 mM, the anodic peaks occurred at around 0.3 V, which was the same as found without glucose. Figure 2B shows the corresponding calibration curve of the current density at oxidation peaks versus glucose concentration; this curve showed linearity until a glucose concentration of 10 mM with a linear regression of 0.985, thereby indicating the good electrocatalytic behavior of the GOx-modified bioanode.

### 3.2. Microfluidic EBFC Characterization for Glucose Biosensing

To check the biosensing ability of microfluidic EBFC, polarization and power density curves were analyzed at various glucose concentrations. An electrochemical method known as chronopotentiometry (CP) was used to obtain the polarization curves, which was accomplished by controlling the current load and attaining variation in output voltage concerning time. Power density curves were plotted by multiplying the current density against the resulting output voltage. The current and power densities were calculated by dividing them by the active surface area of the electrodes. The active surface area of the MWCNT electrodes was 0.4 cm^2^ (with each electrode having an area of 0.2 cm^2^). In the experimental setup, the counter and reference electrode probes of the potentiostat were connected to the bioanode, while the working electrode probe was connected to the biocathode.

**Figure 3:**
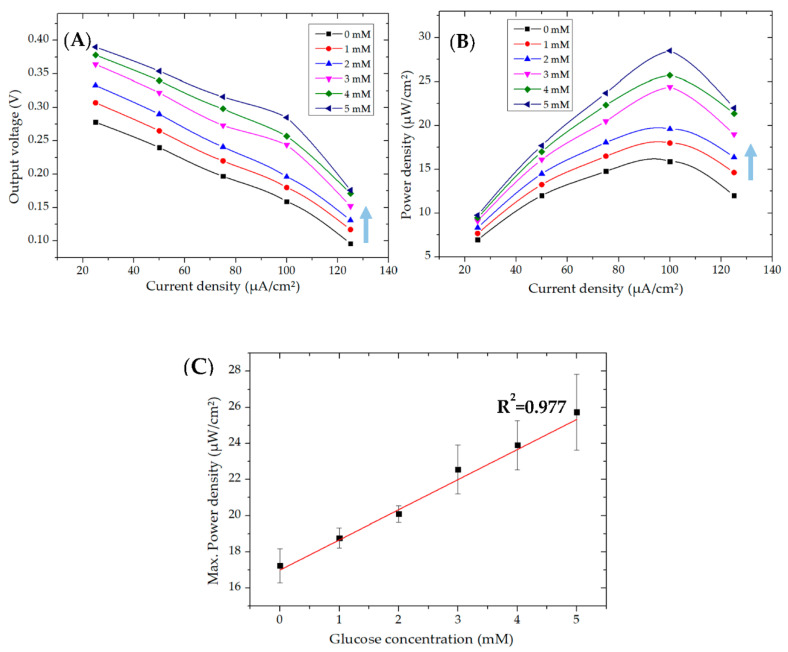
Polarization (**A**) and power density (**B**) curves of a microfluidic EBFC composed of a glucose oxidase (GOx)-attached bioanode and a laccase-attached biocathode operating in different glucose concentrations (0, 1, 2, 3, 4, and 5 mM). The arrows indicate increasing glucose concentration. (**C**) Corresponding calibration curve of the microfluidic EBFC (R^2^ = 0.977).

The polarization and power density curves as a function of glucose concentration are shown in Figure 3A,B, respectively. The results indicated a notable change in the output signal for different glucose concentrations; nonetheless, these curves are not analogous to the previously reported one where different load resistances were applied instead of current loads to obtain the polarization curves [34]. With that method, the power peaks occurred at different current densities for different glucose concentrations. However, contrary to that, it was noticeable that the power peaks occurred at a specific current density of 100 µA∙cm^−2^ for all glucose concentrations, indicating that the use of a single current load was enough to define the calibration curve. Furthermore, it can be seen in Figure 3B that the microfluidic EBFC gave power density even at 0 mM glucose, which was due to the presence of ferrocene methanol in the anolyte solution. This happened as a result of the oxidation of ferrocene methanol to ferrocenium methanol at the anode and the reduction of oxygen to water at the cathode [35]. This phenomenon was also revealed by the CV curve, which is discussed in the previous section in detail. As shown in Figure 3C, the calibration curve concerning peak power density displayed a linear response in the glucose concentration range of 0–5 mM, with a linear coefficient of 0.977. These results assure the capability of the membraneless, microfluidic EBFC to be used as a glucose biosensor.

### 3.3. Effect of the Current Load on the Biosensor Performance

**Figure 4:**
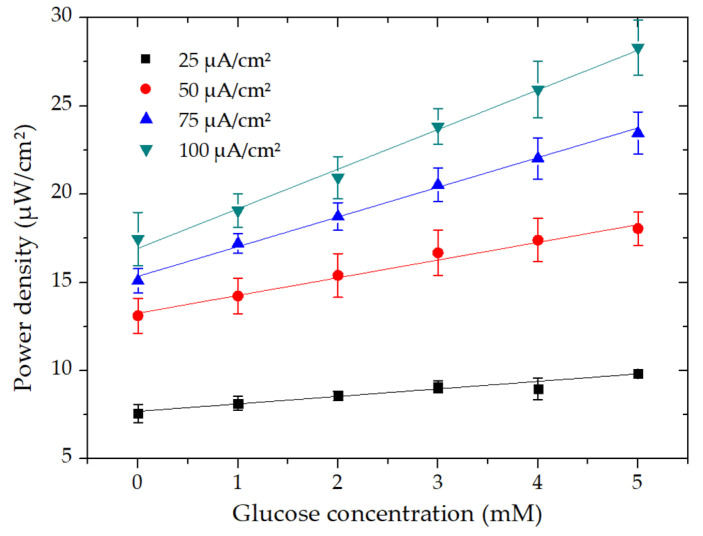
Evaluation of the calibration curves’ sensitivity of the microfluidic EBFC with the addition of glucose concentration concerning different current densities.

As already discussed in the previous section and as is also obvious from the power density curves (Figure 3B), a calibration curve could be obtained at a specific current load. For that reason, the microfluidic chip was evaluated by applying different current loads at glucose concentrations ranging from 0 to 5 mM, as illustrated in Figure 4. The applied current densities were 25, 50, 75, and 100 µA∙cm^−2^. The calibration curves at all the current densities showed linearity until a glucose concentration of 5 mM. It is noteworthy that the slope of the curves got steeper from low to high as current density increased. The sensitivity could be easily calculated from the slope of the linear curve, which was determined as 0.445 and 1.5 µW∙mM^−1^cm^−2^ for 25 and 100 µA∙cm^−2^, respectively. Furthermore, the calibration curve descended with a further increase in current density to 125 µA∙cm^−2^ with linearity up to 4 mM (data not shown). Additionally, it was more convenient to use the resulting voltage as an output signal response instead of power density since the current was maintained at a constant. Nevertheless, the results suggested that a specific current density of 100 µA∙cm^−2^ should be applied for the optimum performance of the microfluidic biosensor.

### 3.4. Effect of Flow Rate on Biosensor Performance

**Figure 5:**
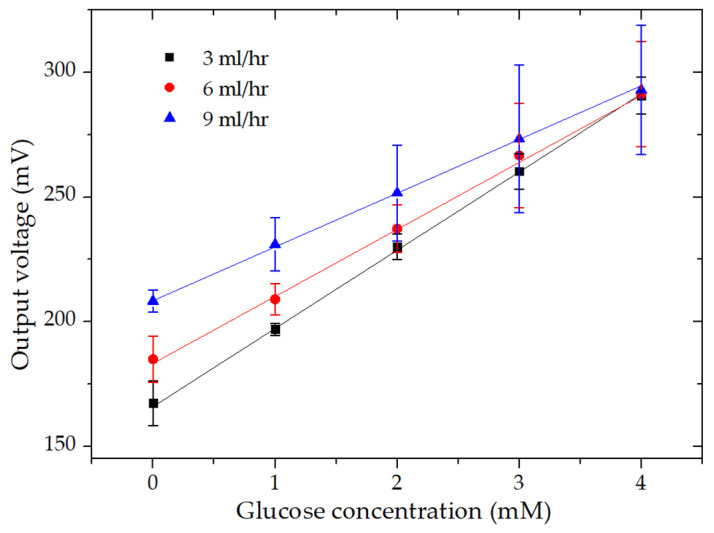
Linearity curves of the output voltage of the microfluidic EBFC at a current density of 100 µA∙cm^−2^ plotted at the flow rates of 3, 6, and 9 mL/h as a function of glucose concentration.

Due to the laminar flow inside the microfluidic channel, the catholyte and anolyte solutions flow in two separate streams in parallel without convective mixing [23]. To optimize the mass transport of fuel and oxidant to their respective biocatalysts, it is necessary to regulate the flow rate of both the electrolytes. For this reason, the membraneless, microfluidic chip was analyzed at different flow rates (3, 6, and 9 mL/h) using glucose concentrations ranging from 0 to 4 mM (Figure 5). Both electrolytes were fed to the microfluidic chip via a peristaltic pump (Reglo digital, Ismatec, Germany). In our setup, the peristaltic pump was used to easily add glucose from the stock solution since we were continuously monitoring glucose concentrations. Before the addition of glucose, the open circuit potential (OCP), which is the maximum output voltage monitored when no current flows in the circuit, was monitored at each flow rate. The OCP values were 436, 410, and 396 mV at the flow rates of 3, 6, and 9 mL/h, respectively (data not shown). From Figure 5, we can see that although the output voltage at a low glucose concentration was lower at 3 mL/h, the output voltage for all three flow rates still reached saturation at a glucose concentration of 4 mM. Hence, we achieved a better sensitivity of 35 mV∙mM^−1^cm^−2^ for the flow rate of 3 mL/h.

### 3.5. Effect of MWCNT Amount on Glucose Determination

**Figure 6:**
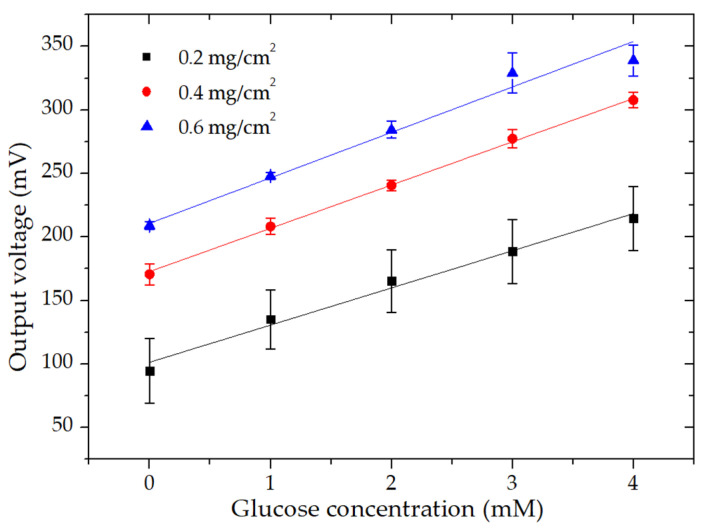
Linearity curves of the output voltage of the microfluidic EBFC as a function of glucose concentration for 0.2, 0.4, and 0.6 mg/cm^2^ MWCNT deposition on electrodes at the current density of 100 µA∙cm^−2^ and the flow rate of 3 mL/h.

Due to its high specific surface area of more than 1000 m^2^/g, MWCNTs are broadly used to provide highly porous nanostructured electrodes [36]. However, the amount of MWCNTs for constructing the electrodes needed to be optimized to achieve a uniform electrode thickness and better performance. The amount of MWCNTs was calculated by the mass of MWCNTs in the solution pipetted per unit surface area of the electrodes. Three samples with different MWCNT amounts of 0.2, 0.4, and 0.6 mg/cm^2^ were prepared as described in Section 2.3. The dependence of the glucose linearity curve as a function of the deposited MWCNT amount is shown in Figure 6. It is worth noting that the optimal MWCNT amount was 0.4 mg/cm^2^ because it gave better linearity and sensitivity. However, when we increased the amount of MWCNTs to 0.6 mg/cm^2^, the electrodes started detaching from the ITO glass in some areas. This little detachment from the ITO glass was due to the drying of the newly pipetted MWCNT solution on the already dried MWCNT layer while on the hot plate. This may happen due to the contraction of the dried MWCNT layer with MWCNT solution during evaporation. This could have been the reason for the small linear range of the calibration curve (i.e., up to 3 mM) at the MWCNT deposition of 0.6 mg/cm^2^.

### 3.6. Detection of Glucose under Optimized Conditions

**Figure 7:**
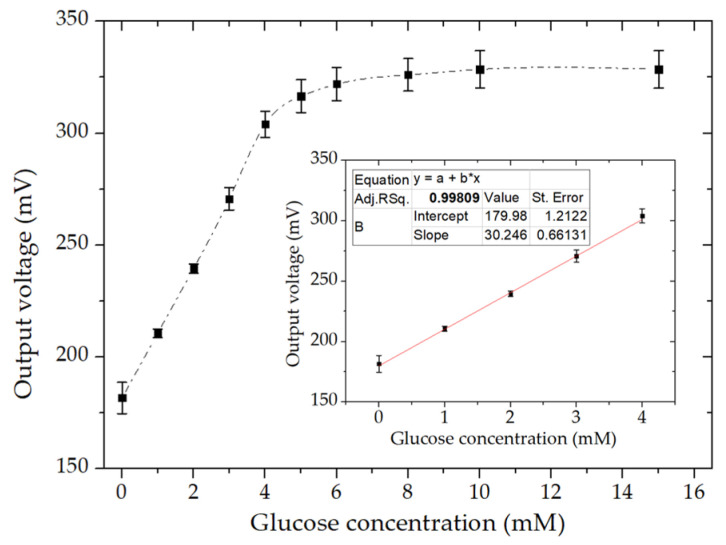
Dependence of the output voltage on glucose concentration for the EBFC based on the GOx-modified bioanode and laccase-modified biocathode under optimized conditions. The output voltage was continuously monitored at an applied current density of 100 µA∙cm^−2^ with the addition of each glucose concentration. The results are shown as the average of the measurements from three devices (mean ± S.D.). Inset: Linear part of the calibration graph.

Figure 7 shows the calibration curve for glucose detection after optimizing several factors, including the MWCNT amount, current loading, and flow rate effect. In the optimized setup, 0.4 mg/cm^2^ MWCNT was deposited on the electrodes, and a current density of 100 µA∙cm^−2^ was applied. The flow rate of the electrolytes was 3 mL/h. The biosensor showed a linear response until a glucose concentration of 4 mM with a linear regression of 0.998. Notably, the calibration curve reached a plateau after 5 mM. This was attributed to the limitation of oxygen reduction at the biocathode, which was a constant that was independent of the increment in glucose concentration [37,38]. It is also clear from the CV results of the GOx-modified bioanode (Section 3.1), where the calibration curve had a linear range until 10 mM of glucose concentration, thus confirming the limitation of performance by the laccase-modified biocathode. The microfluidic EBFC showed a high reproducibility by giving almost the same calibration curves from three different devices. From the error bars, it can be noticed that the results were highly reproducible. The limit of detection (LOD) was 0.23 mM in glucose, which was found using the standard deviation of low concentration (i.e., 3.3 * standard deviation/slope of calibration curve). Moreover, the time constant of the device for sensing the 1 mM glucose concentration was 40 ± 5 s, which was defined as the time required to attain a change from its initial output to 63% of stable output value. The biosensor showed a sensitivity of 31 mV∙mM^−1^cm^−2^, which could be calculated from the slope of the linear calibration curve.

### 3.7. Interference and Recovery Analysis

**Figure 8:**
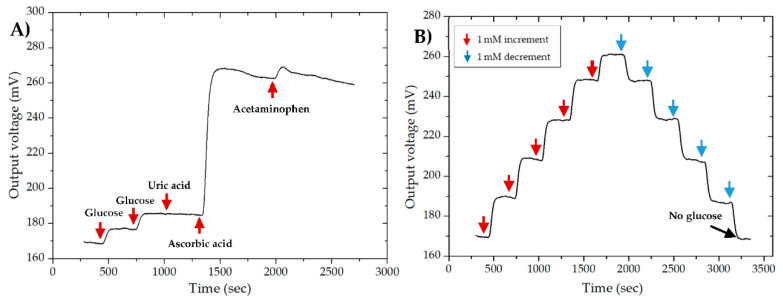
Voltametric responses of the microfluidic, EBFC-based glucose biosensor at a constant current load of 100 µA∙cm^−2^ for (**A**) interference analysis with the stepwise addition of 0.5 mM glucose, 0.3 mM uric acid, 0.1 mM ascorbic acid, and 0.1 mM acetaminophen, as well as for (**B**) recovery analysis with a stepwise 1 mM increment and 1 mM decrement in glucose concentration.

The interferences from electroactive species such as uric acid (UA), ascorbic acid (AA), and acetaminophen (AP), generally found in physiological samples of glucose, were studied. The voltametric responses of the microfluidic, EBFC-based glucose biosensor were obtained at a constant current load of 100 µA∙cm^−2^ with a stepwise addition of 0.5 mM glucose, 0.3 mM UA, 0.1 mM AA, and 0.1 mM AP, as shown in Figure 8A. These amounts of interferents were added according to the minimum of their average concentration in physiological glucose samples. It is noteworthy that there was a slight interference by UA and AP. However, the biosensor showed a significant response upon the addition of AA that was even more than glucose. This result was most probably due to the peroxidation of AA at bioanode since it was the most common compound to oxidize at the same potential range as glucose [39]. This interference by AA could be eliminated using a perm-selective membrane such as a Nafion coating on the enzyme-modified MWCNT electrodes [40,41]. Furthermore, for continuous glucose detection, the recovery characteristics of the device according to stepwise changes in glucose concentration were also recorded, as shown in Figure 8B. The recovery rates were 98.82%, 97.89%, 100.4%, 100%, and 100.5% at glucose concentrations of 0, 1, 2, 3, and 4 mM, respectively.

## 4. Conclusions

This work has demonstrated the feasibility of using membraneless, microfluidic biofuel cells, which were previously fabricated by our group for the electrochemical determination of glucose. The enzymes used as bio-recognition elements were covalently attached to MWCNTs through modification with EDC/NHS. The microchannel was fabricated via PDMS casting, while the electrodes were prepared using a PDMS stencil and MWCNTs patterned on etched ITO glass. The microfluidic device was optimized with different parameters, including flow rate, current load, and MWCNT deposition amount. The enhanced results were obtained at the flow rate of 3 mL/h, a current load of 100 µA∙cm^−2^, and 0.4 mg/cm^2^ of MWCNT deposition on the electrodes. The optimized glucose sensor showed linearity until a glucose concentration of 4 mM, with a linear regression coefficient of 0.998 and a sensitivity of 31 mV∙mM^−1^cm^−2^. Moreover, the microfluidic EBFC also presented good recovery rates, thereby indicating its effectiveness for continuous glucose determination. The proportionality of the power density to the glucose concentration specified the applicability of the proposed EBFC as a self-powered biosensor. This approach of applying a constant current load to simply get the output voltage as an output signal for glucose detection will lead to a simpler design of self-powered glucose sensing systems. However, the low linearity of the glucose calibration curve and the use of mediators in electrolytes were the limitations of this study, and these could be overcome by enhancing the catalytic activity of performance limited cathode and immobilizing the redox mediators on the electrodes, respectively. Though the proposed microfluidic, EBFC-based glucose biosensor has some analytical limitations, the use of such an approach in a simpler and more practical single-flow channel microfluidic device is one of our future goals.
